# Fully automatic evaluation of the corneal endothelium from in vivo confocal microscopy

**DOI:** 10.1186/s12880-015-0054-3

**Published:** 2015-04-26

**Authors:** Bettina Selig, Koenraad A Vermeer, Bernd Rieger, Toine Hillenaar, Cris L Luengo Hendriks

**Affiliations:** Centre for Image Analysis, Uppsala University, Box 337, Uppsala, 75105 Sweden; Rotterdam Ophthalmic Institute, Rotterdam, The Netherlands; Quantitative Imaging Group, Department of Imaging Physics, Delft University of Technology, Delft, The Netherlands; Rotterdam Eye Hospital, Rotterdam, The Netherlands

**Keywords:** Frequency analysis, Stochastic watershed segmentation, Image-based quantification, In vivo imaging, Corneal endothelium

## Abstract

**Background:**

Manual and semi-automatic analyses of images, acquired in vivo by confocal microscopy, are often used to determine the quality of corneal endothelium in the human eye. These procedures are highly time consuming. Here, we present two fully automatic methods to analyze and quantify corneal endothelium imaged by in vivo white light slit-scanning confocal microscopy.

**Methods:**

In the first approach, endothelial cell density is estimated with the help of spatial frequency analysis. We evaluate published methods, and propose a new, parameter-free method. In the second approach, based on the stochastic watershed, cells are automatically segmented and the result is used to estimate cell density, polymegathism (cell size variability) and pleomorphism (cell shape variation). We show how to determine optimal values for the three parameters of this algorithm, and compare its results to a semi-automatic delineation by a trained observer.

**Results:**

The frequency analysis method proposed here is more precise than any published method. The segmentation method outperforms the fully automatic method in the NAVIS software (Nidek Technologies Srl, Padova, Italy), which significantly overestimates the number of cells for cell densities below approximately 1200 mm^−2^, as well as previously published methods.

**Conclusions:**

The methods presented here provide a significant improvement over the state of the art, and make in vivo, automated assessment of corneal endothelium more accessible. The segmentation method proposed paves the way to many possible new morphometric parameters, which can quickly and precisely be determined from the segmented image.

**Electronic supplementary material:**

The online version of this article (doi:10.1186/s12880-015-0054-3) contains supplementary material, which is available to authorized users.

## Background

### Corneal endothelium

The cornea is the frontal, transparent layer of the eye covering the pupil and iris. Its endothelium is composed of a monolayer of hexagonal cells, and plays a pivotal role in the homeostasis of the cornea. Ion exchangers at the basolateral border of the endothelial cells create an osmotic gradient that passively drives corneal fluid towards the anterior chamber. By maintaining the water fraction at 78%, the electrolyte balance ensures corneal transparency. Distortion of this equilibrium caused by damage of endothelial cells leads to swelling of the hydrophilic stroma and a consequent visual loss. In the normal cornea, endothelial cell density slowly declines with age [1]. Several factors such as intraocular surgery or inflammation, however, may speed up the endothelial cell loss. Since the endothelium has very limited regeneration capacity, a decrease in endothelial cell density is compensated by thinning and elongation of the remaining cells. At the same time, the ion exchangers are upregulated to uphold the fluid pumping mechanism [2]. Corneal transparency is affected when the decrease in endothelial cells becomes too large and the pumping mechanism fails. A critical endothelial cell density of 300–500 mm ^−2^ seems needed to prevent corneal decompensation [3]. In ophthalmic practice, this threshold is often used to determine whether or not a patient requires corneal transplantation. The donor cornea, in turn, should have a density of at least 2000 mm ^−2^ to be eligible for keratoplasty. Endothelial cell quantification is also used as a screening method to elect patients for corneal refractive surgery and to determine the rate of endothelial cell loss after iris claw lens implantation.

Even if cell density is the single most important measure when judging the condition of the corneal endothelium, other measures such as variation of cell size and shape are, in principle, also interesting. These measures are, however, not often used in the clinic because of the difficulty in obtaining reliable results. The frequent clinical use of endothelial cell density measurement and its important consequences warrant the development of an objective, fully automatic quantification method. In this paper we introduce two such methods, using images from in vivo white light slit-scanning confocal microscopy. The first simply obtains an estimate of the cell density. The second method produces a delineation of all cells in the field of view. From such a delineation, it is possible to obtain many different morphometric parameters. These methods will enable the use of other morphometric parameters in clinical practice.

### State-of-the-art analysis tools

**Figure 1:**
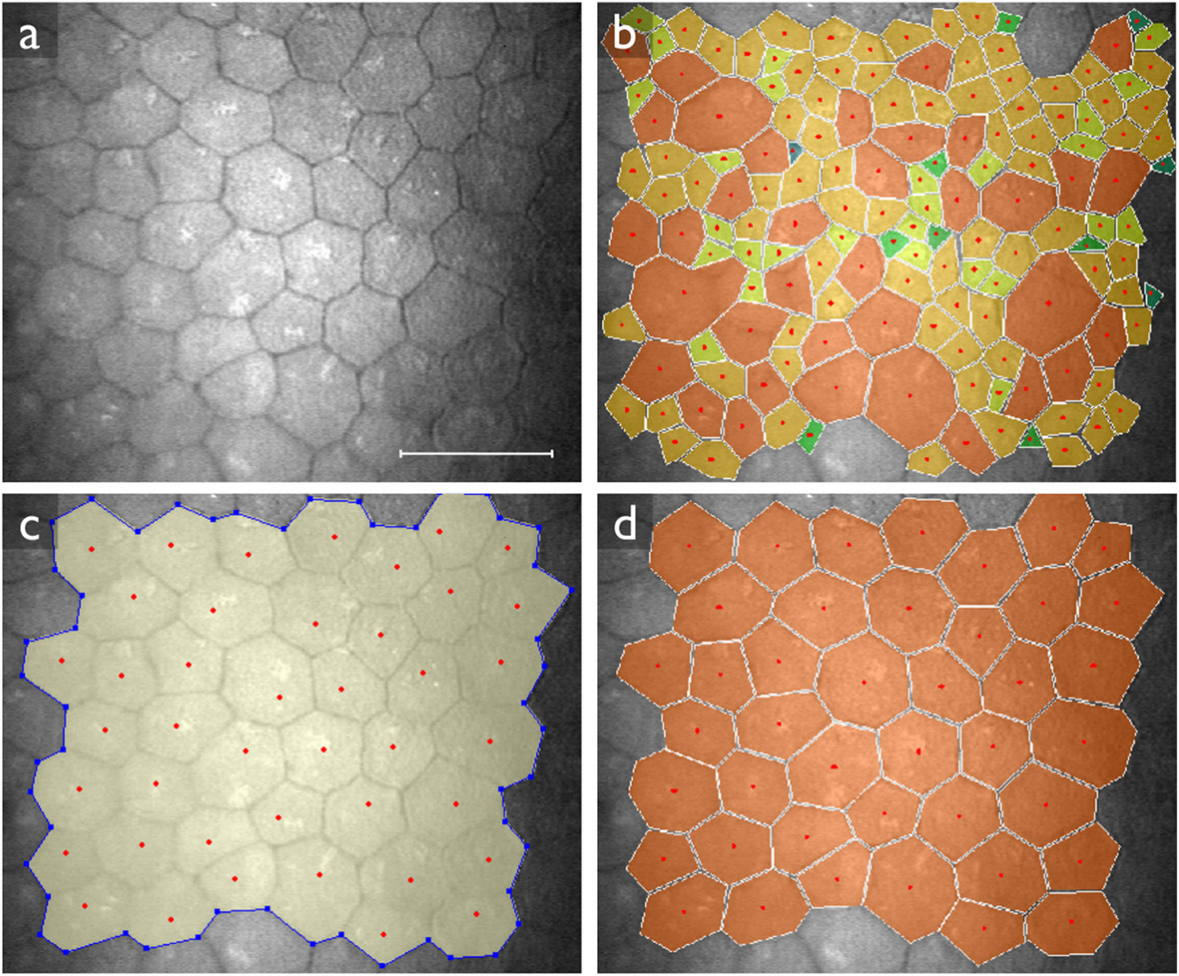
Different endothelial cell count methods performed with NAVIS software on the same confocal image of corneal endothelium after keratoplasty.**(a)** Region of interest. Scale bar = 100 μm. **(b)** Automatic cell count. Endothelial cell density: 2213 mm ^−2^; Polymegathism: 79.0%; Pleomorphism: 33.3%. User interaction time: ≤1 min. **(c)** Polygonal area selection with manual cell count. Endothelial cell density: 687 mm ^−2^; Polymegathism: could not be determined; Pleomorphism: could not be determined. User interaction time: 4 min. **(d)** Manual selection of individual cell borders with manual cell count. Endothelial cell density: 669 mm ^−2^; Polymegathism: 21.5%; Pleomorphism: 52.4%. User interaction time: 20 min.

It is common practice to use commercially available semi-automatic software tools (such as NAVIS by Nidek, IMAGEnet by Topcon, Bambi, Tomey, or EAT by Rhine-Tec) to delineate endothelial cell images and determine morphometric quantities. Even though the procedure is faster and less subjective than manual segmentation, semi-automatic analysis is still tedious and time consuming. The automatic approaches that are commercially available (such as the automatic segmentation mode in the NAVIS software) do not give satisfactory results [4,5] (compare Figures 1b–d).

Methods that automatically estimate the cell density using frequency analysis seem promising [6-9], but the commercial implementations give poor results for both low and high cell densities [10].

Several automatic segmentation methods for corneal endothelium have been proposed recently [11-13]. Of these, only Gavet and Pinoli [11] attempts to solve the problem for images obtained in vivo. These authors use a specular microscope, which produces similar images to those of the confocal microscope used in this paper. The other two papers concern images of in vitro specular microscopy [12] and in vitro inverse contrast phase microscopy [13].

### Frequency analysis

Due to the fairly regular formation of the corneal endothelium (Figure 1), frequency analysis is commonly used to obtain an average cell size. In the late 1980’s and early 1990’s, Masters and colleagues [14-16] observed a concentric ring in the Fourier transform of an image of corneal endothelium. The ring’s radius was related to the average cell size. Some years later, this relation was further explored through the analysis of synthetic images [8,17,18].

The procedure of the most recent approaches to estimate endothelial cell density [6,7,9] is to project the two-dimensional frequency spectrum onto a one-dimensional function. One salient spatial frequency on this function is then found, often a peak. This frequency, which we will here call the *characteristic frequencyf*^⋆^, is related to the average cell size, and thus to cell density. We review these methods, and propose a new one, under the heading “Frequency analysis” in the “Methods” section.

All published approaches make the same, implicit assumption *A*=(*f*^⋆^)^−2^ about the relationship between *f*^⋆^ and the average cell area *A*. This is, in general, not correct, and under the heading “Density estimation” in the “Methods” section we show how to establish the correct relationship.

### The stochastic watershed algorithm

For more complex analyses of the endothelial layer, a full segmentation of the cells is needed. In confocal images of the endothelial layer, the cells are visualized as bright areas separated by dark lines (Figure 1). An excellent method to segment such images is the watershed algorithm [19,20]. This algorithm segments the image into regions with one local minimum each. The segmentation lines run along the brightest line in between each pair of local minima. Thus, the confocal images of the endothelium must be inverted first, such that the cell boundaries are bright lines. Unfortunately, due to the noise in the image, each cell typically has many local minima, and therefore will be segmented into many regions by the watershed algorithm. One solution is to apply the H-minima transform before the watershed algorithm [21]. This transform suppresses all local minima that have a depth less than the parameter *h*. However, it is often impossible to find a value *h* such that each cell has exactly one local minimum.

An alternative to avoid oversegmentation is to apply a seeded watershed [22]. One seed point needs to be placed in each cell; a minimum imposition algorithm will transform the image such that these seeds will be the only local minima in the image. Consequently, the watershed algorithm will generate a single region for each seed. The logic required to automatically place one seed point in each cell is far from trivial, and thus this method is often used with some manual interaction.

In the stochastic watershed approach, the seeded watershed is repeatedly applied to the image with randomly placed seeds [23]. The segmentation results are added together to build a map that shows the likelihood of each pixel belonging to a boundary. This map is referred to as probability density function, PDF. More salient boundaries will have a larger value in the PDF. A segmentation is obtained from this map by applying a smoothing, the H-minima transform, and finally the classical watershed algorithm.

Bernander et al. [24] proposed two modifications to the stochastic watershed algorithm to greatly increase its ability to distinguish salient boundaries and reduce oversegmentation. The segmentation method proposed under the heading “Segmentation” in the “Methods” section is based on this version of the stochastic watershed.

## Methods

### Materials

We used a set of 52 confocal images of corneal endothelium. These images were acquired in 23 patients with Fuchs’ endothelial corneal dystrophy using a white light slit-scanning confocal microscope (Confoscan 4; Nidek Technologies Srl, Padova, Italy). All patients were examined in the first year after Descemet stripping automated endothelial keratoplasty.

Because the diseased endothelium was replaced by donor endothelium, the images varied considerably concerning the morphometric parameters, but did not contain corneal guttae, characteristic of Fuchs’ endothelial corneal dystrophy. Before the examination, the eyes were anesthetized with one drop of 0.4% oxybuprocaine (Ceban BV; Breda, The Netherlands). We used a 40 × objective lens together with a z-ring adapter to minimize the eye movements during the 12 seconds of image acquisition. A coupling gel (Vidisic; Dr. Mann Pharma, Berlin, Germany) was applied between the objective lens and the corneal surface. All scans were performed using fixed device settings: full-thickness mode, 72% light intensity, and a 6 μm scan step. The images were captured with a charge-coupled device camera, producing 768 ×576 pixels and 8-bit integer pixel values; we manually cropped each image to remove the dark areas, leaving only the well-illuminated central part. The cropped images had a mean area of 219146 px^2^ (range [124740,400575]). According to the manufacturer, the lateral sampling density was 0.557 μm per pixel.

In each of these images, an expert marked a set of cells using the NAVIS software (Nidek Technologies Srl, Padova, Italy). He placed a point in the middle of each cell and drew an outline around the set of cells (polygonal frame method [25]). Thus, in the outlined region (evaluation area *A*_eval_) is a known number of complete cells *n*_eval_. We consider the morphometric quantities that were then determined by the NAVIS software as ground truth. We also used the fully automatic segmentation mode of the NAVIS software to obtain a second set of measurements for comparison.

Data was collected in accordance with the tenets of the Declaration of Helsinki and informed consent was obtained from all participants. Approval was obtained from the Medical Ethical Committee of the Erasmus Medical Center, Rotterdam, The Netherlands (METC-2007-131). All data is freely available at http://rod-rep.com.

### Frequency analysis

Below, we review published methods to derive the characteristic frequency from the frequency spectrum of the image, and propose a new one.

#### Radial mean of the frequency spectrum

The standard method to obtain an orientation-independent representation of the frequency content of a two-dimensional image is to compute the radial mean of the magnitude of its Fourier transform,

(1) $$ \mathcal{F}_{\text{RM}}(f) = \frac{1}{2\pi} \int_{0}^{2\pi} |\mathcal{F}(f,\theta)| ~d\theta \quad,  $$

where $\mathcal {F}(f,\theta)$ is the Fourier transform of the image in polar form, i.e. *f* is the radial frequency and *θ* the angle. To compute the radial mean, we follow this procedure: First, we define the output to be sampled at frequencies $f=\left \{0,\frac {1}{N},\frac {2}{N},\ldots,\frac {1}{2}\right \} \text {px}^{-1}$, given *N* the width of the image in pixels (assuming a square image). Next, we determine the frequency for each sample in the frequency spectrum, and round the value to the nearest frequency in the list above. Finally, we compute the mean of all values assigned to the same frequency.

The mode of $\mathcal {F}_{\text {RM}}$ is the characteristic frequency *f*^⋆^, and is related to the characteristic length *λ* of the structures in the image by *λ*=1/*f*^⋆^. We determine the position and strength of all local maxima in $\mathcal {F}_{\text {RM}}$ by fitting a parabola to three consecutive points around each local maximum, excluding the one at *f*=0. The position of the local maximum with the highest value is the characteristic frequency. We refer to this method as *modeRM*.

Bucht et al. [9] take the first peak in $\mathcal {F}_{\text {RM}}$ as the characteristic frequency. We refer to this method as *1stRM*.

#### Radial maximum of the frequency spectrum

Ruggeri et al. [7] proposed to compute the radial maximum of the magnitude of the Fourier transform,

(2) $$ \mathcal{F}_{\textrm{RMAX}}(f) = \max_{\theta \in [0,2\pi)} |\mathcal{F}(f,\theta)| \quad.  $$

They observed two peaks in this function, and stated that the first peak was related to the slow variation in the image intensity and the second peak represented the frequency of the repetitive cell pattern. Therefore, they suggested to use the second peak of $\mathcal {F}_{\textrm {RMAX}}$ as characteristic frequency. We compute the radial maximum in an analogous way to the radial mean, and determine the precise location of the peak by fitting a parabola. We refer to this method as *2ndRMAX*.

#### Radial mean of the power spectrum

Foracchia and Ruggeri [6] used the mode (*modePS*) of the radial mean of the power spectrum,

(3) $$ \mathcal{F}_{\text{PS}}(f) = \frac{1}{2\pi} \int_{0}^{2\pi} |\mathcal{F}(f,\theta)|^{2} ~d\theta \quad.  $$

They also discuss using the mean (*meanPS*) or median (*medianPS*) instead. We compute the radial projection of the power spectrum in an analogous way to the radial projections above. However, Foracchia and Ruggeri [6] computed the cumulative radial mean by integrating the frequency spectrum over increasingly large circles, then computed the derivative of this cumulative distribution. Depending on the method used to determine the derivative, this results in either the same or a very similar function to that obtained by our implementation. They also applied a smoothing to $\mathcal {F}_{\text {PS}}$ before computing the mode, mean or median. The size parameter of this smoothing operator was not given in their paper, but presumably has a very strong effect on the result, especially in the case of the mode. We chose to not apply any smoothing. We compute the mode of $\mathcal {F}_{\text {PS}}$ in the same way as in the method modeRM.

The methods meanPS and medianPS require the application of a high-pass filter to the image. We implement this by setting the first *N* entries of the function $\mathcal {F}_{\text {PS}}$ to zero. We indicate this parameter by appending it to the method name: *meanPS 3*, *medianPS 5*, etc.

#### Enhancing the ring prior to computing the radial mean

This paragraph proposes a new method, based on modeRM.

On occasions when the modeRM method fails at determining the correct cell density, we observed that the peak of $|\mathcal {F}(f,\theta)|$ at *f*=0 decays very slowly, and drowns the ring that we want to detect. We therefore developed a simple procedure to completely remove the central peak from the frequency spectrum before computing the radial mean.

The procedure applies the dilation by reconstruction [26] of the pixel at *f*=0 with $|\mathcal {F}|$ as mask. That is, the image

(4) $$ H_{0}(f,\theta) =\left\{ \begin{array}{ll} |\mathcal{F}(0,\theta)| & ~~\text{for}~~ f=0 \\ 0 &~~ \text{otherwise} \end{array} \right.  $$

is iteratively dilated, until stability, under the constraint that the result be smaller than $|\mathcal {F}|$ after every iteration:

(5) $$ H_{t} = \left(H_{t-1} \oplus B \right) \wedge |\mathcal{F}| \quad.  $$

The structuring element *B* used has the smallest possible shape: the origin and its four direct neighbors. In practice, this operation is very fast due to a priority-queue algorithm that addresses each pixel exactly once [27]. The result of the operation, $H_{\infty }$, is the central peak with its long tail, and without the ring. The function $H_{\infty }(f,\theta)$ is decreasing for increasing *f* and constant *θ*. This would not be the case if a larger structuring element *B* was used.

**Figure 2:**
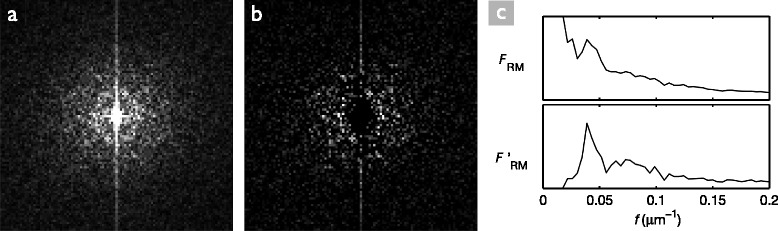
Enhancement of the central ring.**(a)** Magnitude of the frequency spectrum $|\mathcal {F}|$ for a typical image; only the central part is shown. **(b)** The image $\mathcal {F}'$; the central peak has been removed, which enhances the ring. **(c)** Radial mean projections for $|\mathcal {F}|$ (top) and $\mathcal {F}'$ (bottom); note that the peak corresponding to the characteristic frequency is much more salient in the bottom graph. The vertical scaling in the two graphs is not the same.

The image $\mathcal {F}' = |\mathcal {F}| - H_{\infty }$ contains the ring, but not the central peak. Note that this process is equivalent to a high-pass filter, very specifically constructed for the image at hand. Any other high-pass filter (such as a Gaussian high-pass filter) would have a parameter to be tuned, as is the case in the meanPS and medianPS methods. See Figure 2 for an example of the ring enhancement and how it affects the radial mean projection.

The method proposed here will be referred to as *modeRMrec*; it computes the radial mean of $\mathcal {F}'$, and finds the position of the maximum by fitting a parabola.

### Density estimation

If *f*^⋆^ is the characteristic frequency determined by one of the methods above, then *l*=1/*f*^⋆^ is related to the most common cell width. The average area *A* of one cell can now be calculated by

(6) $$ A = \alpha l^{2} = \frac{\alpha}{{f^{\star}}^{2}} \quad,  $$

where *α* is a factor that depends on the shape and regularity of the cells.

**Figure 3:**
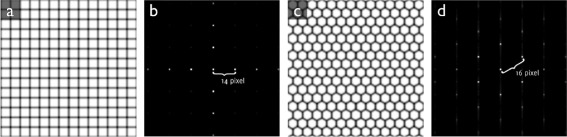
Relation between cell shape, characteristic frequency, and cell density. Synthetic, band-limited images (350×350 pixels) of **(a)** square and **(c)** hexagonal cell pattern with 25 pixel side-to-side length for each cell. **(b, d)** Magnitude of the central region of the respective frequency spectra. Characteristic frequencies are $\frac {14}{350}=\frac {1}{25}\textit {px}^{-1}$ and $\frac {16}{350}\approx \frac {2}{\sqrt {3}}\frac {1}{25}\textit {px}^{-1}$, respectively.

Figure 3 shows two synthetic, band-limited images of 350 ×350 pixels with a hexagonal and a square cell pattern; in both images, each cell has a side-to-side length of 25 pixels. The same figure also shows the magnitude of the frequency spectrum of these two images. For the square pattern, the characteristic frequency $f^{\star } = \frac {1}{25}\;\textit {px}^{-1}$, and the area of each cell *A*=25^2^ px^2^, leading to *α*=*Af*^⋆^^2^=1. However, for the hexagonal pattern, $f^{\star } = \frac {2}{\sqrt {3}} \frac {1}{25}\;\textit {px}^{-1}$, $A = \frac {\sqrt {3}}{2}25^{2}\;$px^2^, and thus $\alpha = \frac {2}{\sqrt {3}} \approx 1.15$. Note that the hexagonal packing is the most dense possible. Thus, in practice, one would expect *α*<1.15. Note also that there is no reason to expect *α*≥1, as irregular patterns are likely to have lower values.

Previous publications have not considered the effect of the cell shape on the relationship between characteristic frequency and cell density. Except mentioning that “the geometrical pattern [of corneal endothelial cells] is not regularly hexagonal” [6], no explicit assumption of the cell shape is made [7,9]. Therefore, it seems that all these authors made the implicit assumption *α*=1.

Assuming that the cell density of the imaged section is representative for the whole endothelium, the endothelial cell density can be calculated with

(7) $$ \delta_{f^{\star}} = \frac{1}{\alpha} {f^{\star}}^{2} \quad.  $$

### Segmentation

In this section, we propose a new method to fully automatically segment the corneal endothelium. The method is based on the stochastic watershed, as described in the introduction. This algorithm has two steps. First, the seeded watershed is applied *m* times to the inverted input image. For each of these repetitions, uniformly distributed noise in the range [0,*u*] is applied to the image, and *n*_seeds_ seeds are placed randomly. The results of these *m* segmentations are added together, yielding a PDF. In the second step, the PDF is blurred with a Gaussian filter with parameter *σ*_PDF_, local minima are removed by a H-minima transform with parameter *h*, and then the classical watershed algorithm is applied. Optionally, the resulting segmentation can be refined to better match the location of the cell boundaries.

**Figure 4:**
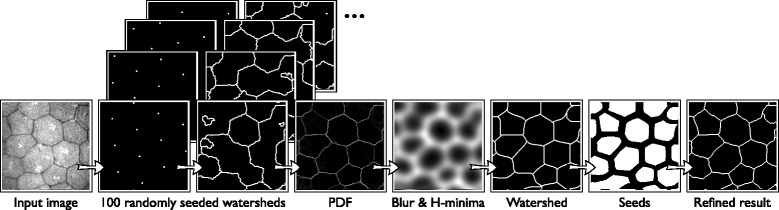
Schematic representation of the segmentation procedure, as detailed in the “Methods” section.

The procedure is described in detail below, including the methodology to determine the optimal values for all parameters, given a collection of images with a known ground truth. Refer to Figure 4 for a schematic representation of the segmentation procedure.

#### Determining the number of seed points *n*_seeds_

Ideally, *n*_seeds_ is equal to the number of regions in the segmented image. We can estimate this number using the cell density estimate we obtained through frequency analysis. As will be shown in the results section, the modeRMrec method is the most suitable one to determine the characteristic frequency *f*^⋆^. Given a total image area of *A*_*I*_, the number of cells expected to be found in the image is given by

(8) $$  n_{\text{seeds}} = A_{I} \delta_{f^{\star}} = \frac{1}{\alpha} A_{I} {f^{\star}}^{2} \quad.  $$

The value of *n*_seeds_ is typically an underestimation of the number of regions that needs to be segmented, because some of the cells are intersected by the image border. Nonetheless, the stochastic watershed as used here is not very sensitive to the number of seeds [24,28], so it is acceptable to use this value. We may assume *α*=1 at this stage for the same reason.

#### Computing the stochastic watershed

To compute the stochastic watershed, we apply *m*=100 [24] repetitions of the seeded watershed with randomly placed seeds. To place the seeds, we take a hexagonal grid with edge length

(9) $$ t = \sqrt{\frac{2}{3\sqrt{3}}\,\frac{A_{I}}{n_{\text{seeds}}}} \approx \frac{1}{f^{\star}}\sqrt{\frac{2}{3\sqrt{3}}} \quad,  $$

give it a random translation and rotation, and use the vertices as seed locations [24]. The given edge length *t* creates a seed density equivalent to the cell density.

At each repetition of the seeded watershed, we add uniformly distributed noise in the range [ 0,*u*] to the image. This noise randomizes the order in which the pixels are processed, avoiding the reinforcement of spurious segmentation lines [24]. The results of all seeded watershed segmentations are added together to create a PDF, an image where pixels have a value between 0 and *m*, indicating how often each pixel was chosen to be part of a region boundary. Pixels chosen more frequently are more likely to be part of an actual cell boundary.

Due to the varying noise and the width of the boundary between cells, it is likely that the same boundary was slightly shifted in various repetitions of the seeded watershed. To unify these boundaries, we apply a smoothing filter with a Gaussian kernel. The optimal size *σ*_PDF_ of this kernel depends on the cell size, so we define a parameter *k*_*σ*_=*σ*_PDF_*f*^⋆^ that is independent of the cell size.

Next, we apply the H-minima transform with a parameter *h*=*k*_*h*_*m*/*σ*_PDF_, to discard small minima. Here, *k*_*h*_ is the parameter to be optimized, the fraction makes this value independent of *m* and *σ*_PDF_. Finally, the classical watershed algorithm segments the image into individual cells.

As we will see later, the computation of the PDF is not greatly affected by a wrong estimation of the value of *f*^⋆^. In contrast, the smoothing of the PDF is rather sensitive to changes in this estimate. To have a more consistent smoothing, it is possible to estimate *f*^⋆^ again from the PDF. We will study this improvement under the heading “Influence of estimated cell density on the segmentation algorithm” of the results section

#### Correcting segmentation borders

The segmentation obtained by the stochastic watershed follows ridges in the image smoothed by a Gaussian kernel with size *σ*_PDF_. Such a smoothing tends to shift edges and ridges. Hence, for large *σ*_PDF_, the ridges found are displaced with respect to those in the input image. This final, optional step corrects the delineation and improves subsequent cell shape measurements.

We first shrink all segmented regions by 20%, producing a marker image

(10) $$ S(x) =\left\{ \begin{array}{ll} 1 & ~~\text{if}~~ \frac{D_{\mathrm{c}}(x)}{D_{\mathrm{c}}(x) + D_{\mathrm{b}}(x)} < 0.8 \\ 0 & ~~\text{otherwise} \end{array} \right. \,,  $$

where *D*_c_(*x*) and *D*_b_(*x*) are the Euclidean distance to the center of mass and to the boundary of the region, respectively. Next we slightly smooth the inverted, original image with a Gaussian kernel with *σ*=2. Finally, we compute the seeded watershed of this slightly smoothed image using the marker image *S* as seeds.

By shrinking the segmented regions only slightly, we avoid large shifts of the boundaries. The purpose of this last step is not to find new boundaries, only to better align them with the boundaries in the unsmoothed image.

#### Evaluating the segmentation

The ground truth for the data set used consists of a point (marker) near the middle of each cell in the evaluation region (*n*_eval_ markers). The evaluation region was manually outlined as described in the materials section. Because it is hand-drawn, this outline cannot perfectly match the delineation by the segmentation algorithm, and thus we need to allow for small deviations.

We consider a cell correctly segmented if it contains exactly one ground-truth marker, and lies for at least 85% inside the evaluation region. We ignore any segmented regions outside of the evaluation region, as well as any region that overlaps the evaluation region by less than a quarter of the average region size. Using these definitions, we can determine *n*_total_, the total number of regions found, and *n*_corr_, the number of correctly segmented regions.

We then compute the precision *p*=*n*_corr_/*n*_total_ and the recall *r*=*n*_corr_/*n*_eval_, and combine them into the *F*-measure

(11) $$ F = 2 \frac{p r}{p + r} \quad.  $$

Following this definition, oversegmented cells decrease the precision *p* and under-segmented cells decrease the recall *r*. Because *F* is the harmonic mean of *p* and *r*, lowering either of them lowers *F*.

#### Parameter training

The segmentation method as presented here depends on 3 parameters: *u* (noise range), *k*_*σ*_ (blur size), and *k*_*h*_ (local minima to ignore).

To determine the best values for these parameters, given a set of training images with known ground truth, we apply the algorithm to each image in the set with different values for the three parameters. We then evaluate every resulting segmentation, determining the *F*-measure as described in the previous section, and compute the average (mean) *F* for each combination of parameters. The set of parameters that yields the highest average *F* is the optimal set.

### Morphometry

The health condition of the endothelial layer can be determined using three morphometric quantities: cell density, polymegathism (cell size variability) and pleomorphism (cell shape variation). Cell density is defined as the inverse of the mean cell size; polymegathism is defined as the coefficient of variation of the cell size, and typically expressed as a percentage; and pleomorphism is defined as the percentage of cells that are hexagonal, which we compute by counting the percentage of cells with six neighbors. To avoid measuring partially imaged objects, we discard all segmented regions touching the image border.

### Method evaluation

We evaluate our methods using leave-one-out cross validation [29]. Given a total of *M* images for both training and testing, we train the method with *M*−1 images, and test on the remaining image. This process is repeated *M* times, such that each image has been used once for testing. The *M* test results are then averaged.

## Results

### Density estimation

**Figure 5:**
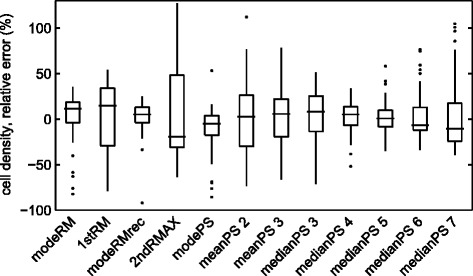
Relative error in estimated cell density for the various frequency analysis methods, as standard box plots. The box indicates the interquartile range, the line inside the box indicates the median, the whiskers indicate the extrema, and the dots indicate outliers.

We tested the various frequency analysis methods by computing the endothelial cell density and comparing that with the manually estimated values. We used leave-one-out cross validation to determine an appropriate *α* for each image and each method. Figure 5 plots the relative errors.

The methods meanPS and medianPS have one parameter *N* (for the high-pass filter). We illustrate results with *N*∈{2,3} for meanPS and *N*∈{3,4,5,6,7} for medianPS, larger or smaller values produced worse results. With smaller values of *N*, these methods produced values of *f*^⋆^ very close to zero, which obviously does not indicate any relevant frequency in the image. Thus, the high-pass filtering is indispensable for these methods.

The narrowest distributions of relative errors are obtained by the methods modeRM, modeRMrec, modePS and medianPS 5. Of these four, modePS and modeRM have the widest distributions and the most outliers. modeRMrec has a slightly narrower distribution and fewer outliers than medianPS 5, but it has one extreme outlier. Furthermore, the method medianPS 5 has a parameter that might need to change if the imaging setup changes; note that we selected this parameter using the same data set as we used to test the method.

Values of *α* for the modeRMrec method were in the range 0.97–0.99.

### Training parameters of the segmentation algorithm

The proposed segmentation algorithm has 3 parameters for which optimal values need to be determined: the noise range *u*, the smoothing size *k*_*σ*_, and the local minima depth *k*_*h*_. To avoid influence of the chosen frequency analysis method in the parameter training, we use a number of seeds given by the ground truth,

(12) $$ n_{\text{seeds}} = A_{I} \frac{n_{\text{eval}}}{A_{\text{eval}}} \quad.  $$

**Figure 6:**
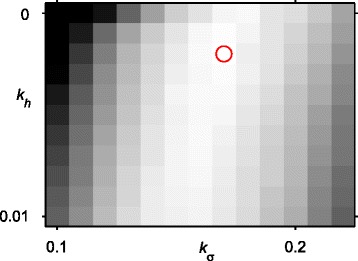
Mean *F*-measure for all images in the data set, with *u*=30 and different values of *k*_*σ*_ and *k*_*h*_. Black indicates *F*≤0.8, white is for *F*=0.9142, and occurs at *k*_*σ*_=0.17 and *k*_*h*_=0.002 (marked by the red circle); this is the global maximum. This same combination of parameters were found to be optimal in each of the cases during leave-one-out cross validation.

We then ran the segmentation algorithm on all images in the data set for all values of *u* between 20 and 50 in steps of 10, values of *k*_*σ*_ between 0.10 and 0.22 in steps of 0.01, and values of *k*_*h*_ between 0.000 and 0.010 in steps of 0.001. We calculated the *F*-measure for each result. We then determined parameters to be used for each image *i* as follows: we took all the images in our set excluding image *i*, computed the mean *F* over these images for each combination of parameters, and determined the combination of parameters that yielded the largest average *F*. We will refer to these values as *u*_*i*_, *k*_*σ*,*i*_ and *k*_*h*,*i*_. Testing the algorithm using these parameters is analogous to leave-one-out cross validation. For all images *i*, the optimal parameters were the same: *u*_*i*_=30, *k*_*σ*,*i*_=0.17 and *k*_*h*,*i*_=0.002. The parameter space in all these cases was smooth and convex (see Figure 6). The mean F-measure over all images was 0.907.

We also tested the method by Gavet and Pinoli [11] (using code provided by the author), as well as a much older method by Vincent and Masters [30]. We had to smooth and correct illumination of the input image to the latter method, since it was designed for in vitro images of a much lower resolution, with less noise and fewer artefacts. These methods had four and three parameters, respectively, which were optimized in the same way as described above. Vincent and Masters [30] provided the better results, with a mean F-measure of 0.821. Gavet and Pinoli [11] yielded a mean F-measure of 0.722. We did not have direct access to the segmentation results of the automatic mode in the NAVIS software, so were not able to compute *F*-measures, but the computed cell densities lie somewhere in between those of the methods by Vincent and Masters [30] and Gavet and Pinoli [11].

### Stochasticity of the segmentation

The segmentation algorithm proposed here is stochastic, meaning that it can produce a different result every time it is run on the same image. We studied the influence of the stochasticity on the result by looking at the variation in *F*-measure over 12 segmentations of each image, using the optimal parameters found in the previous section. The interquartile range (IQR) of *F* varied from image to image, between 0 and 0.074. This indicated that for some images most segmentations were identical, whereas for other images the quality varied somewhat. In general, the images that yielded a larger average *F* also had a smaller variation in *F*, with a clear inverse correlation between the median and the range of *F* (*ρ*=−0.78).

Note that increasing the number of repetitions *m* of the seeded watershed in the stochastic algorithm will reduce the variation of the result, but not improve the results. Using the four images in the set that had the median *F* closest to the median over all images, we repeated the segmentation with increasing *m*, and observed the reduction in the range of obtained *F*-measures. The average *F* for these four images did not change significantly as *m* increased.

### Influence of estimated cell density on the segmentation algorithm

The cell density $\phantom {\dot {i}\!}\delta _{f^{\star }}$ is used to determine the number of seeds for the segmentation algorithm. A rough estimate is obtained using the frequency analysis method. The following experiment supports our claim that the segmentation algorithm is very robust against changes in the number of seeds.

We applied the segmentation algorithm to each of the images *i*, using parameters *u*_*i*_, *k*_*σ*,*i*_ and *k*_*h*,*i*_ as before, but varied the number of seeds between 25% and 400% of the optimal value as determined from the ground truth. The results are shown in Table 1 (Setup 1). Note how the mean *F*-measure for the segmentations does not change significantly as *n*_seeds_ changes by a factor of 2 in either direction. This shows that it is not problematic to assume *α*=1 in the segmentation algorithm.

**Table 1:** **Effect of the density estimation on the proposed segmentation algorithm**

***n***_**seeds**_	**Corresponding** ***f*** ^**⋆**^	**Setup 1**	**Setup 2**	**Setup 3**
**(fraction of**	**(fraction of**	**mean** ***F***	**mean** ***F***	**mean** ***F***
**optimum)**	**optimum)**			
0.25	0.50	0.883	0.206	0.874
0.50	0.71	0.907	0.766	0.908
0.75	0.87	0.926	0.913	0.923
1.00	1.00	0.914	0.914	0.915
1.50	1.22	0.928	0.892	0.925
2.00	1.41	0.918	0.791	0.910
4.00	2.00	0.831	0.465	0.807

*F*-measures are averaged over all 52 images. Setup 1: only *n*_seeds_ changes. Setup 2: *f* ^⋆^ changes, affecting both *n*_seeds_ and *σ*_PDF_. Setup 3: As in Setup 2, but *f* ^⋆^ is re-calculated from the PDF to determine *σ*_PDF_.

The characteristic frequency *f*^⋆^ also influences the size *σ*_PDF_ of the smoothing filter. If we do not only change the number of seeds, but change the value of *f*^⋆^ correspondingly according to Equation 8 (simulating what would happen if the frequency analysis produced a wrong result), the smoothing filter will change as well, affecting the segmentation result significantly. This case is reported as Setup 2 in Table 1.

One approach to reduce the influence of the frequency analysis on the segmentation algorithm is to apply modeRMrec again on the PDF produced in the stochastic watershed, and use its result to determine *σ*_PDF_. We tested this procedure and reported it as Setup 3 in Table 1. Note that the average *F*-measures are now much less influenced by the initial estimate *f*^⋆^.

### Morphometry

**Figure 7:**
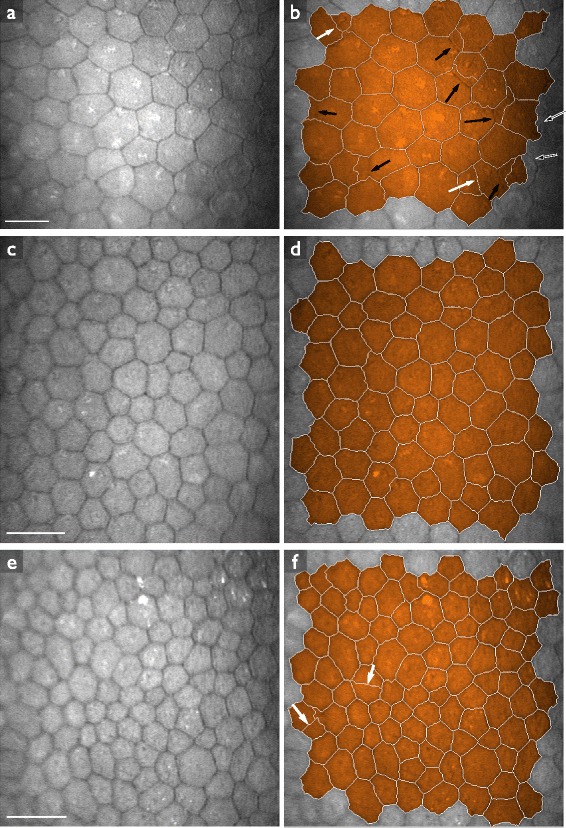
Example results of the fully automatic segmentation algorithm.**(a)** The image used in Figure 1, and **(b)** the segmentation result. This is an endothelium with a low cell density, for which the NAVIS automatic method failed (compare with Figure 1b and d). The result of the proposed method produced a reasonable result, with only two cells too many (white arrows) and a few misplaced cell boundaries (black arrows). **(c)** One of the images for which the segmentation **(d)** had a perfect score. **(e)** A typical high-density endothelium, for which the segmentation **(f)** only had two oversegmented cells. Scale bars = 50 μm.

**Figure 8:**
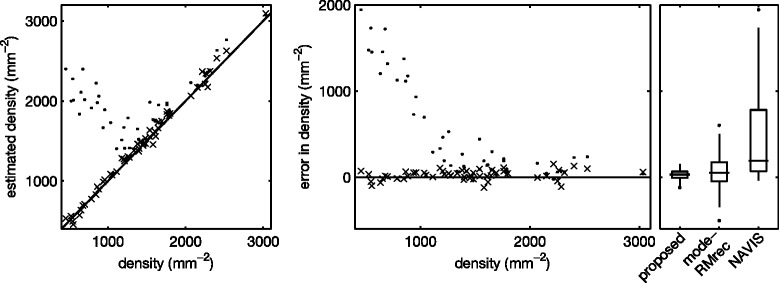
Cell density. Estimated cell density (left) and error in estimated cell density (right), compared with the manual ground truth, for the segmentation method proposed here (×) and the NAVIS software in fully automatic mode (·). Note that the NAVIS software has an error that depends on the cell density. To the right, standard box plots summarize the results. The error of the modeRMrec method is included for comparison.

**Figure 9:**
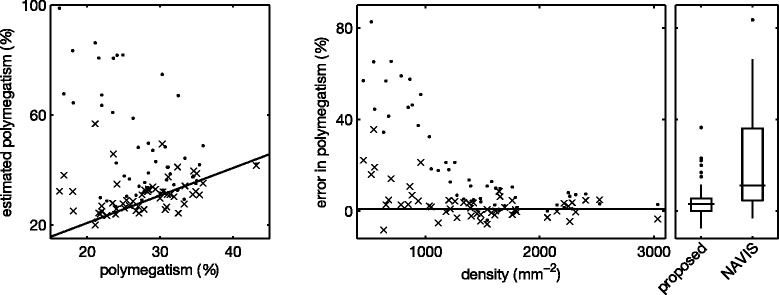
Polymegathism. Estimated polymegathism (left) and error in estimated polymegathism (right), compared with the manual ground truth, for the segmentation method proposed here (×) and the NAVIS software in fully automatic mode (·). To the right, standard box plots summarize the results.

**Figure 10:**
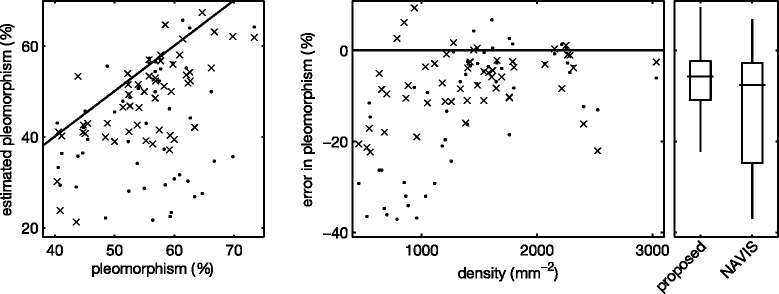
Pleomorphism. Estimated pleomorphism (left) and error in estimated pleomorphism (right), compared with the manual ground truth, for the segmentation method proposed here (×) and the NAVIS software in fully automatic mode (·). To the right, standard box plots summarize the results.

Next, we again applied the segmentation algorithm to each of the images in the set, using the optimal parameters *u*_*i*_, *k*_*σ*,*i*_ and *k*_*h*,*i*_ obtained through leave-one-out cross validation. We estimated *f*^⋆^ through the modeRMrec method, and re-estimated it from the PDF before applying the smoothing filter, as suggested in the previous section. Some example segmentation results are shown in Figure 7. From the segmentations we determine the morphometric quantities (cell density, polymegathism and pleomorphism) as described in the “Methods” section. We compared these measurements with the ground truth, and plotted the results in Figure 8, 9 and 10. These figures also contain the results obtained by the automatic mode in the NAVIS software. Estimated values for each image can be found in Additional file 1: Table S1. We can observe an overall improvement compared to the NAVIS software, especially for images with a cell density below ∼ 1200 mm^−2^. Figure 8 also includes cell density estimates obtained through frequency analysis (modeRMrec), and shows that the segmentation yielded a much improved result. Note that, especially for low cell densities, images have few cells, and the slightly different choice of cells measured between manual and automatic method can have a large effect on estimated quantities.

## Discussion

### Density estimation

We introduced the factor *α* to determine the endothelial cell density based on frequency analysis. The value of *α* depends on the shape of the repeating pattern in the image. For a perfect hexagonal pattern *α*≈1.15, as illustrated in Section “Density estimation”. However, for the less uniform pattern of the typical endothelium, the value for *α* is on average closer to 1. This is presumably the reason previous authors were able to make the implicit assumption of *α*=1 [6-9].

**Figure 11:**
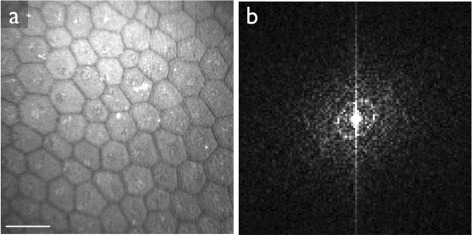
Example image where the cell pattern has a strong directional preference (panel **a**). The ring in the frequency spectrum (panel **b**) is elliptic. Scale bar = 50 μm.

We observed that the cell structure as seen in the images can have a strong directional preference (Figure 11). In these cases, the ring in the frequency spectrum is elliptic rather than circular. The elliptic ring yields a wider peak in the one-dimensional radial projection, and leads to a mode that is a rough approximation of the average radius of the ring. In extreme cases, the peak could become so wide as to not be salient enough for detection. Nonetheless, the results on the data set used in this paper did not warrant specifically addressing this issue.

**Figure 12:**
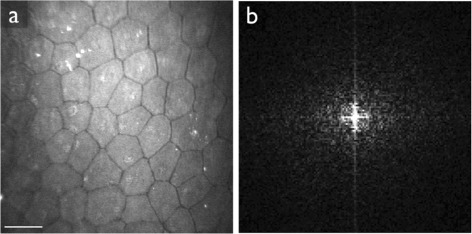
The only image in data set where the reconstruction by dilation step in modeRMrec fails (panel **a**). In (panel **b**), magnitude of the corresponding frequency spectrum. Scale bar = 50 μm.

The modeRMrec method proved to be the most precise of the frequency analysis methods, and does not have any parameters to tune. However, for one image from the data set, the modeRMrec method completely failed to detect the ring (see the point close to -100% in Figure 5). Figure 12 shows this image and the magnitude of its frequency spectrum. When applying the dilation by reconstruction algorithm, most of the ring was removed, causing the peak for the correct *f*^⋆^ to disappear. The estimated *f*^⋆^ was 4 times smaller than it should be. A larger data set is needed to know how common this effect is, and whether software should be able to detect or correct for this situation.

A problem with two of the frequency analysis methods we studied, meanPS and medianPS, is that they employ a high-pass filter. We implemented the high-pass filter by discarding a set number of low-frequency bins from the radial projection. The number of bins to discard, i.e. the parameter to the high-pass filter, needs to be determined from example images. In our tests, the medianPS approach gave very good results when discarding the first five bins (medianPS 5). However, the method is very sensitive to changes in this parameter, as can be seen from the results for medianPS 4 and medianPS 6 in Figure 5. Other ways of implementing a high-pass filter would allow a more fine-grained tuning of the filter’s parameter.

The NAVIS software has problems segmenting corneal endothelium with low cell density, as seen in Figures 8, 9 and 10, and previously mentioned by [4]. In Figure 8, we can observe that the automatic method strongly overestimated the density in images with a cell density below ∼1200 mm ^−2^. In fact, the software never produced a density estimate below 1000 mm ^−2^. One possible explanation for this phenomenon could be that the method by Foracchia and Ruggeri [6], which was implemented in the NAVIS software, does not use frequencies below 0.05 px ^−1^, which corresponds to a cell density of 939 mm ^−2^ ([6], Figure 5). That is, any density below 939 mm ^−2^ yields a peak in the frequency spectrum below 0.05 px ^−1^, and would not be found by the NAVIS software. A peak at a higher frequency, but close to this cutoff, would be affected as well. Given that 300–500 mm ^−2^ is a critical density for the endothelium to uphold its fluid pumping function, this cutoff is puzzling.

### Segmentation

The proposed segmentation method uses *f*^⋆^ to determine a suitable number of seed points *n*_seeds_ for the stochastic watershed, and to determine the parameter *σ*_PDF_ to the smoothing applied to the PDF. The experiments in Section “Influence of estimated cell density on the segmentation algorithm” show that the influence of *f*^⋆^ on *n*_seeds_ is small for the final segmentation, but its influence on *σ*_PDF_ is significant. To reduce the influence of a poor frequency analysis result on the final segmentation, we recommend to repeat the frequency analysis step on the PDF produced in the stochastic watershed. This PDF is a clean representation of the cell boundaries, in which neither the non-uniform illumination nor the other details are visible. Consequently, the frequency analysis of the PDF yields a much more precise estimate of *f*^⋆^, and is less likely to be influenced by imaging artifacts.

The two steps used to simplify the PDF reduce the number of local minima. However, they both do this in different ways. The H-minima transform (directed by the parameter *k*_*h*_) directly removes local minima, without affecting other areas of the PDF. In contrast, the smoothing (directed by the parameter *k*_*σ*_) changes the appearance of the PDF by widening and straightening the lines. As can be seen in Figure 6, these two parameters are strongly related, as changing one of them changes the optimal choice for the other. Both these operations are needed, as the global optimal combination has non-zero values for both parameters; however, the H-minima transform is the least important, as it is possible to obtain an average *F* close to the optimal with *k*_*h*_=0.

The segmentation method is fully automatic, meaning that no user intervention is required to segment a given image into individual cells. However, there are three parameters that need to be tuned. We derived optimal values for these parameters given data from a specific instrument and a manually drawn ground truth. This procedure might need to be repeated if the method is to be applied to images from a different instrument. However, once these values have been optimized, it is not necessary to tweak these values for individual images.

Manually-drawn ground truth is often problematic when training and testing segmentation algorithms, because they are never perfect. Different experts will draw outlines differently, and will not always agree on diagnoses. However, because of the way we define and use the ground truth in our experiments, we can be quite certain that a different expert’s ground truth will not produce different *F*-measures: the data we used does not pose difficulties for an expert determining the extent of cells, and we allow small variations in the outline drawn around the selected cells. The only difference we have seen with repeated creation of a ground truth for an image is in which cells are contained in the evaluation region.

The segmentation algorithm proposed here is stochastic in nature. That means that it can produce a different result every time it is applied to the same image. In principle, after $m=\infty $ repetitions, the result is deterministic. This is, of course, impractical, and so *m*=100 has been chosen in line with the recommendation by Bernander et al. [24]. As was discussed in Section “Stochasticity of the segmentation”, increasing *m* does not improve the average *F*, even if it reduces its range. This implies that the probability for both extremely poor as well as extremely favorable segmentations is reduced. That is, increasing *m* can increase the confidence in the segmentation result. However, *m* is directly proportional to the time it takes the algorithm to produce its result. Hence, a compromise has to be made between precision and computation time. *m*=100 has been deemed as an acceptable compromise [24]. Malmberg and Luengo Hendriks [31] have recently shown that it is possible to efficiently compute the PDF of the stochastic watershed for $m=\infty $ repetitions. However, this algorithm can only work for the case *u*=0 (i.e. without the noise added for every repetition), which produces very poor results in this application.

### Morphometry

It is common procedure to judge the condition of the corneal endothelium based on cell density, polymegathism and pleomorphism. However, it is unclear how reliable these measurements are. The main issue is that only a small fraction of the endothelium is visible in one image, and there exists no test to confirm whether the region imaged is representative of the whole endothelium [13], meaning that the results have an unknown precision.

Furthermore, some authors advised caution when comparing measurements obtained by different methods [4,5,32], indicating that different methods introduce different biases. Having a fast, fully automatic method as presented in this paper would allow recording more images, to cover a larger fraction of the endothelium, and thereby guaranteeing more precise morphometric estimates. However, this does not solve the problems with possible biases.

Of the three morphometric quantities, pleomorphism is the one that is most affected by errors in the segmentation. This is because each under- or over-segmented cell causes several neighboring cells to have a wrong polygon edge count. Furthermore, both errors of over- or under-counting polygon edges cause the pleomorphism measure to increase; these errors do not compensate for each other. Given that this measure is so unreliable, it might be worth considering alternative measures for the regularity of the endothelium, such as cell compactness or elongation, which can be computed on each cell independently.

Polymegathism has larger errors for very low density (Figure 9), as a segmentation error in that case affects the estimate more. In contrast, pleomorphism has similar errors at all densities (Figure 10).

Figure 8 shows a slight bias in the density estimation from the segmentation. One possible cause could be the way that cells are selected for this estimate: we discard cells partially in the image, which causes a bias towards smaller cells because large cells are more likely to be cut by the image border. This source of bias is carefully studied in the field of stereology, and can be solved using a counting frame [33]. We have not applied this method because the field of view in these images is rather small, with few cells imaged. Using a counting frame would further reduce the number of cells counted.

Bucht et al. [8] analyzed the frequency spectrum of a large number of simulated cell patterns. They concluded that the ratio of amplitudes of the center and the first harmonic peaks is related to the variation of cell sizes. In our notation, the center peak amplitude corresponds to the D.C. value, $\mathcal {F}_{\text {RM}}(0)$ (equal to the mean grey value of the image), and the first harmonic peak amplitude corresponds to $\mathcal {F}_{\text {RM}}(f^{\star })$. Unfortunately, this relationship only holds for their synthetic images. As Bucht et al. [8] noted, larger cell variation causes the peak at *f*^⋆^ to be smeared out, making it wider. In the idealized synthetic images they used, this translated to a lower peak as well. However, in real-world images, several other factors also affect the height of this peak as well as that of the center peak, such as the contrast, uneven illumination, noise, etc. Furthermore, to measure the width of a peak is not trivial in these data, and we have not found a reliable method to estimate the cell size variation using frequency analysis only.

## Conclusions

In this paper we have introduced an algorithm that can automatically analyze the corneal endothelium, as imaged using in vivo confocal microscopy, with a reasonable accuracy. In some images, the algorithm’s result may need further refinement. A simple and quick correction could be performed by the operator, as typically only a few cells need to be merged or split. With the right user interface, such a correction could take less than half a minute, yet yield a wealth of accurate morphometric parameters of the endothelium; currently an operator needs four minutes to simply estimate the cell density. Morphometric parameters other than density have not been practical in a clinical setting up to now, though polymegathism and pleomorphism are used in some situations. The segmentation method presented in this paper paves the way for a broader use of such parameters in the clinic. Now that it is easy to obtain a segmentation of the cells in the corneal endothelium, it will be interesting to study which morphometric parameters are important to monitor in various diseases. Measures like compactness (a ratio of the perimeter and the area of each cell) and elongation are easily quantified, and might provide better quality measures.

## Additional file

Additional file 1
**Values of manual and automatic estimates of endothelial parameters for each of the images in our data set.** We include the automatic estimates from the NAVIS software and from the segmentation algorithm proposed in this paper.
