# Supplementary material for: Fully automatic evaluation of the corneal endothelium from in vivo confocal microscopy
Source: BMC Med Imaging. 2015 Apr 26;15:13. doi: 10.1186/s12880-015-0054-3 (PMC4451752; doi:10.1186/s12880-015-0054-3)
Supplement: Additional file 1 — Values of manual and automatic estimates of endothelial parameters for each of the images in our data set. We include the automatic estimates from the NAVIS software and from the segmentation algorithm proposed in this paper. [file 12880_2015_54_MOESM1_ESM.pdf]

Table S1 Values of manual and automatic estimates of endothelial parameters for each of the images in our data set. We include the automatic estimates from the NAVIS software and from the segmentation algorithm proposed in this paper.

image size		ground truth				NAVIS			proposed method			
x (px)	y (px)	cells	density (mm^{-2})	polymegathism (%)	pleomorphism (%)	density (mm^{-2})	polymegathism (%)	pleomorphism (%)	density (mm^{-2})	polymegathism (%)	pleomorphism (%)	F-measure
378	476	65	1541	33.3	56.9	1634	29.3	56.5	1553	27.6	52.3	0.99
491	416	71	1396	25.8	66.2	1523	36.7	50.0	1368	27.0	55.2	0.93
353	354	52	1760	43.2	40.4	1949	42.2	43.1	1831	41.6	30.2	0.94
390	553	85	1799	30.0	44.7	1862	31.5	36.5	1845	29.7	41.1	0.92
351	415	78	2152	23.9	57.7	2196	26.6	57.0	2169	23.8	58.1	0.98
349	456	91	2215	27.5	62.6	2193	29.1	64.0	2367	30.9	54.3	0.91
411	448	97	2285	35.0	41.2	2359	36.0	36.4	2174	38.7	40.2	0.86
538	476	59	958	16.8	59.3	1890	67.7	22.5	1014	38.1	40.4	0.85
561	553	63	883	18.1	69.8	2059	64.4	35.7	897	25.0	62.1	0.90
491	516	53	848	22.0	58.5	2222	67.3	29.5	830	24.9	64.7	0.91
420	466	83	1760	28.4	62.7	1974	38.9	44.2	1870	33.1	52.3	0.95
403	525	65	1204	29.0	53.8	1668	47.1	34.2	1274	33.8	42.6	0.89
346	475	123	3033	34.5	45.5	3073	37.4	39.5	3094	31.0	43.0	0.92
351	476	84	2068	34.9	52.4	2230	35.0	49.5	2064	32.1	49.4	0.97
408	463	64	1481	30.9	40.6	1441	36.4	33.3	1531	26.5	41.2	0.95
431	476	74	1485	26.7	54.1	1522	30.8	51.3	1465	25.2	46.5	1.00
445	476	84	1540	33.4	50.0	1982	44.3	45.5	1635	34.2	39.0	0.89
420	448	111	2264	35.4	57.7	2248	41.9	55.0	2346	30.9	56.8	0.94
349	471	58	1653	31.1	44.8	1950	41.0	42.1	1737	37.5	42.6	0.90
441	456	80	1608	31.9	48.8	1746	34.6	55.6	1618	30.2	43.0	0.93
543	415	73	1381	29.4	57.5	1649	43.0	52.3	1464	32.9	41.6	0.90
498	476	64	1112	30.7	60.9	1404	48.4	31.7	1116	25.6	58.1	0.94
403	468	78	1693	30.4	52.6	1723	32.7	48.7	1746	32.0	46.8	0.95
438	476	82	1615	31.0	45.1	1761	41.0	45.7	1559	31.1	40.8	0.93
455	470	67	1276	27.1	52.2	1412	34.0	52.0	1296	24.2	54.0	0.99
525	476	54	1034	26.3	66.7	1730	58.8	34.7	1085	28.6	63.2	0.97
538	476	57	937	23.5	43.9	1666	60.9	35.8	989	27.9	53.3	0.96
535	470	64	1050	24.0	73.4	1087	42.5	64.2	1074	25.8	61.9	0.99
575	476	42	699	18.0	59.5	2016	83.4	23.4	708	32.1	50.0	0.90
638	533	55	677	22.0	56.4	2398	63.5	21.7	694	26.9	38.5	0.89
496	373	63	1421	25.4	54.0	1490	30.4	50.7	1493	29.0	51.6	0.98
490	416	70	1452	21.1	61.4	1471	21.7	65.7	1441	20.0	61.5	0.99
493	353	95	2252	21.8	55.8	2312	29.9	56.3	2209	24.0	57.0	0.97
393	425	103	2526	34.6	59.2	2763	37.8	46.2	2625	39.6	37.2	0.84
421	420	102	2316	31.4	59.8	2382	38.6	56.3	2371	31.1	56.0	0.98
485	473	104	1788	22.7	55.8	1884	28.7	57.3	1816	23.4	53.4	0.95
511	476	81	1400	27.8	56.8	1514	32.1	54.4	1404	25.6	53.0	0.98
521	501	51	786	21.6	64.7	1912	80.7	27.6	772	24.3	67.4	0.93
369	476	74	1645	32.4	56.8	1735	32.6	50.0	1664	41.1	48.6	0.89
353	476	48	1179	27.0	58.3	1512	48.2	37.3	1285	26.7	51.2	0.94
349	476	72	1579	35.9	51.4	1768	48.8	47.9	1458	35.2	46.6	0.83

(continues)

Table S1 (continued).

image size		ground truth				NAVIS			proposed method			
x (px)	y (px)	cells	density (mm^{-2})	polymegathism (%)	pleomorphism (%)	density (mm^{-2})	polymegathism (%)	pleomorphism (%)	density (mm^{-2})	polymegathism (%)	pleomorphism (%)	F-measure
433	516	63	1257	28.4	52.4	1787	49.7	28.1	1303	32.7	41.2	0.91
520	476	53	863	24.1	62.3	1978	81.7	30.3	926	34.8	51.9	0.89
456	476	33	653	24.9	48.5	2108	81.8	22.2	636	27.7	40.0	0.83
610	543	41	526	16.2	63.4	2000	98.9	26.9	561	32.2	42.1	0.79
583	555	35	456	23.6	60.0	2399	80.6	30.8	530	45.8	39.5	0.86
763	525	39	554	30.3	43.6	2008	74.8	29.0	457	49.5	21.3	0.63
491	476	40	633	32.5	55.0	1836	67.0	28.7	571	24.2	50.0	0.86
595	555	44	546	21.1	40.9	2276	86.3	29.4	496	56.8	23.8	0.43
324	385	76	2404	27.9	55.3	2633	35.4	43.0	2534	32.6	39.2	0.85
476	476	65	1216	25.1	52.3	1408	37.8	39.0	1250	26.1	51.5	0.93
405	520	71	1335	28.3	62.0	1403	33.6	55.2	1379	32.0	53.6	0.94
